# An Intracellular Arrangement of *Histoplasma capsulatum* Yeast-Aggregates Generates Nuclear Damage to the Cultured Murine Alveolar Macrophages

**DOI:** 10.3389/fmicb.2015.01526

**Published:** 2016-01-11

**Authors:** Nayla de Souza Pitangui, Janaina de Cássia Orlandi Sardi, Aline R. Voltan, Claudia T. dos Santos, Julhiany de Fátima da Silva, Rosangela A. M. da Silva, Felipe O. Souza, Christiane P. Soares, Gabriela Rodríguez-Arellanes, Maria Lucia Taylor, Maria J. S. Mendes-Giannini, Ana M. Fusco-Almeida

**Affiliations:** ^1^Faculdade de Ciências Farmacêuticas, UNESP – Univ Estadual Paulista, Campus Araraquara, Departamento de Análises Clínicas, Laboratório de Micologia ClínicaSão Paulo, Brazil; ^2^Departamento de Microbiologia y Parasitologia, Facultad de Medicina, Universidad Nacional Autónoma de MéxicoMéxico City, México

**Keywords:** *Histoplasma capsulatum*, host-pathogen interactions, intracellular arrangement, nucleus, alveolar macrophages

## Abstract

*Histoplasma capsulatum* is responsible for a human systemic mycosis that primarily affects lung tissue. Macrophages are the major effector cells in humans that respond to the fungus, and the development of respiratory disease depends on the ability of *Histoplasma* yeast cells to survive and replicate within alveolar macrophages. Therefore, the interaction between macrophages and *H. capsulatum* is a decisive step in the yeast dissemination into host tissues. Although the role played by components of cell-mediated immunity in the host's defense system and the mechanisms used by the pathogen to evade the host immune response are well understood, knowledge regarding the effects induced by *H. capsulatum* in host cells at the nuclear level is limited. According to the present findings, *H. capsulatum* yeast cells display a unique architectural arrangement during the intracellular infection of cultured murine alveolar macrophages, characterized as a formation of aggregates that seem to surround the host cell nucleus, resembling a “crown.” This extranuclear organization of yeast-aggregates generates damage on the nucleus of the host cell, producing DNA fragmentation and inducing apoptosis, even though the yeast cells are not located inside the nucleus and do not trigger changes in nuclear proteins. The current study highlights a singular intracellular arrangement of *H. capsulatum* yeast near to the nucleus of infected murine alveolar macrophages that may contribute to the yeast's persistence under intracellular conditions, since this fungal pathogen may display different strategies to prevent elimination by the host's phagocytic mechanisms.

## Introduction

Many studies have been performed to elucidate the interaction between the dimorphic fungus *Histoplasma capsulatum* and host macrophages, specifically to determine the role played by the components of the host's cell-mediated immunity and the evasion mechanisms used by the pathogen. In some conditions, in contrast to their usual function of eliminating deleterious microorganisms, macrophages give rise to a favorable environment for the survival and reproduction of the *H. capsulatum* yeast phase, which is the parasitic-virulent morphotype of this fungus (Medeiros et al., 2002; Tagliari et al., 2012). *H. capsulatum* has been described as a facultative intracellular pathogen, and it is almost exclusively found within host-parasitized cells (Wu-Hsieh et al., 1998; Hilty et al., 2008). Once the pathogen has been phagocytosed, several immunological factors can modulate the course of the infection (Allen and Deepe, 2005).

According to Newman et al. (2011), the destruction of alveolar macrophages and their subsequent ingestion by other immune cells are events that promote the propagation of the infection to different organs during the acute stage of primary histoplasmosis. Thus, it is clear that the interaction between the macrophage and *H. capsulatum* is a decisive step in the occurrence of yeast dissemination into host tissues.

Apoptosis of phagocytes in the initial stage of infection by *H. capsulatum* activates CD4+ and CD8+ T cells, both of which partially act as a defense mechanism for the host. Hsieh et al. (2011), described that apoptosis induced by the infection is an important immune function recognized by the antimicrobial host response mainly in the defense against phagosome-enclosed pathogens. Hence, inhibition of apoptosis modulates the inflammatory response and also interferes in the outcome of the infection process. According to Allen and Deepe (2005), IL-4 and IL-10 production are enhanced when apoptosis is inhibited, with the release of these cytokines exacerbating the fungal infection. This result occurs because the apoptosis of macrophages, which is induced early in a pulmonary infection by *H. capsulatum*, releases IL-10, which inhibits apoptosis of neighboring macrophages, enabling and delimiting the intracellular residence of *H. capsulatum* yeast (Deepe and Buesing, 2012).

Nuclear fragmentation is a morphological cellular alteration associated with apoptosis (Deepe and Buesing, 2012); thus, nuclear damage in host cells can be characterized as a cellular effect that contributes to the pathogenesis of histoplasmosis. Glukhov et al. (2008) reported that bacterial endotoxins induce nuclear DNA damage in human mononuclear cells, which is associated with the infectious process and disease manifestation. However, knowledge of the DNA fragmentation induced by microorganisms is limited. Hence, it is necessary to investigate the behavior of nuclear envelope proteins during infection.

Nuclear envelope proteins promote a functional link between support structures, cytoplasmic compartments, and nucleoplasmic compartments. These proteins have been identified as components of the LINC complex (nucleoskeleton and cytoskeleton linker), which are specific to the outer and inner nuclear membranes. The LINC complex is composed primarily of SUN nuclear proteins and by Nesprin, although other envelope proteins, such as Emerin, can also be identified. An illustration of the LINC complex organization can be found in Haque et al. (2010). These proteins play important roles in the positioning, migration and maintenance of nuclear architecture (Ostlund et al., 2009; Taranum et al., 2012). In addition, this complex is critically important because the blade and associated proteins play a role in modulating gene expression (Martins et al., 2012).

In general, Nesprin binds to actin and several other motor proteins of the microtubule network. In the inner nuclear membrane, SUN-domain proteins (SUN1, SUN2, and SUN3) bind to the blade in the nucleoplasm. Moreover, the Emerin protein characterized as a transmembrane protein may be associated with microtubules in the outer nuclear membrane, and can bind to the nuclear lamina when it is located in the inner nuclear membrane. Thus, the LINC complex passes through the perinuclear space and connects the components of the cytoskeleton with the nuclear lamina (Crisp et al., 2006; Martins et al., 2012). The labeling of nuclear envelope proteins in host cells could contribute to the characterization of the behavior of these proteins during the course of an *in vitro* infection.

The mechanisms by which *H. capsulatum* interacts with macrophages and evades host immune defenses have been well documented. However, this is the first report that attempts to characterize the interaction pattern and the nuclear damage of parasitized host cells after the internalization of *H. capsulatum* yeast in order to verify the correlation of these yeast cells with host cell integrity. Apoptosis assays were performed as well as the staining of nuclear envelope proteins in host cells infected with the fungus. Our study highlights the intracellular behavior and the effects induced by *H. capsulatum* at a nuclear level in cultured infected alveolar macrophages.

## Materials and methods

### Fungal growth conditions

*H. capsulatum* strains EH-315 and 60I were used. The EH-315 strain was isolated from a naturally infected bat and was deposited in the *H. capsulatum* Culture Collection of the Fungal Immunology Laboratory of the Department of Microbiology and Parasitology, from the School of Medicine, National Autonomous University of Mexico (UNAM) (www.histoplas-mex.unam.mx). This collection is registered in the database of the World Federation for Culture Collections under the number LIH-UNAM WDCM817 (www.wfcc.info/ccinfo/index.php/collection/by_id/817/). The 60I strain was isolated from a human clinical case and was deposited in the collection of the Clinical Mycology Laboratory of the Faculty of Pharmaceutical Sciences, UNESP, Brazil. Yeasts were grown in brain–heart infusion (BHI-broth) (Difco Laboratories, Detroit, MI, USA) and supplemented with 0.1% L-cysteine and 1% glucose, at 37°C, for 24 h, and with rotary agitation (100 rpm). Dispersed *H. capsulatum* yeast cells were washed three times with phosphate-buffered saline (PBS), followed by low-speed centrifugation for 1 min at 600 × *g* to remove large yeast clumps. Suspensions of single yeast cells were separated for counting with a hematocytometer.

### Macrophage cultures

Murine alveolar macrophages, AMJ2-C11 cell-line, were cultured overnight at 37°C on coverslips placed in the well-bottom of 24-well plates (TPP®, Trasadingen, Switzerland) using Dulbecco's modified Eagle's medium (DMEM) (Sigma-Aldrich, St Louis, MO, USA) supplemented with 10% heat-inactivated fetal calf serum (Cultilab, Campinas, SP, Brazil).

### Ethics statement

Rabbits were used for antibody production. They were processed exactly as outlined in the experimental protocol recommended by the Ethics Committee on Animal Experiments of the Faculty of Pharmaceutical Sciences of Araraquara—UNESP (reference number: 10/2011/CEUA/FCF), which was approved for this study. All efforts were made to minimize suffering in all animal procedures.

### Immunoglobulin to cell-free antigen of *H. capsulatum*

*H. capsulatum* cell-free antigen, a rich solution of cell wall associated antigens, was prepared as described previously by Sá-Nunes et al. (2005). Protein concentration was quantified using the Bradford method (BioRad Laboratories Inc., Hercules, CA, USA). To prepare a polyclonal antibody raised against cell-free antigen of *H. capsulatum*, rabbits were inoculated by intradermal injection of 1.0 mL of the cell-free antigen mixed with 1.0 mL of complete Freund's adjuvant. Subsequent injections of this antigen with incomplete Freund's adjuvant were given weekly for a period of 4 weeks, and thereafter monthly, for a period of 3 months. The rabbits were bled at the 7th day after the last dose. The immunoglobulin fraction of each rabbit anti-serum was separated by precipitation with ammonium sulfate and stored at −70°C.

### Infection rate of *H. capsulatum* in alveolar macrophages detected by colony forming units (CFU)

For this assay, a reference strain from the American Type Culture Collection (ATCC), G-217B, was compared with strains EH-315 and 60I. The infection rate of each strain was estimated using the AMJ2-C11 alveolar macrophage cell-line (ATCC, CRL-2456). The assay was performed in 24-well plates (TPP®) containing 10^5^ AMJ2-C11 macrophages per well, as described by Sardi et al. (2012). Each cultured macrophage monolayer was infected with 500 μL of yeast inoculum (1 × 10^6^ yeasts/mL) and plates were incubated at 37°C for 0, 7, 15, 30, 60, 120, 180, and 300 min (5 h). After each incubation time, a macrophage monolayer was washed three times with sterile PBS to remove released yeast cells. Then, the AMJ2-C11 cells were detached at 37°C for 2 min using trypsin-EDTA (Gibco Life Technologies, Carlsbad, CA, USA) diluted in PBS. Subsequently, 100 μL of each infected macrophage suspension was plated on supplemented BHI-agar (Difco) and incubated at 37°C, for 24–72 h. After incubation, fungal colonies were counted and the CFU/mL was estimated for each strain tested, corresponding to the number of *H. capsulatum* yeast cells that was able to infect the alveolar macrophage monolayer at each incubation time. For each assay, a control for yeast cell viability was performed in which yeast cells were maintained with trypsin-EDTA for 2 min and Trypan blue solution was added afterward to detect viability. *H. capsulatum* infection rate curves were constructed based on the data of each strain incubated at the different times. Tests were set up in triplicate in two independent assays.

The interaction between alveolar macrophages and *H. capsulatum* yeast were also monitored by conventional Giemsa staining and indirect fluorescence.

### Indirect immunofluorescence

Samples of infected macrophages were maintained under the optimal culture conditions for a 5 h incubation. The infected monolayers were fixed with 4% paraformaldehyde, washed in PBS, and permeabilized in 0.5% Triton X-100 for 30 min. Polyclonal anti–*H. capsulatum* antibody was added for a 1 h incubation at room temperature, and unbound antibodies were removed by washing with PBS. Alexa Fluor®594-conjugate goat anti-rabbit IgG (Invitrogen-Molecular Probes, Eugene, OR, USA) was added and incubated for 1 h at room temperature and, subsequently, fluorescein isothiocyanate (FITC)-labeled phalloidin (Sigma-Aldrich, St Louis, MO, USA) was added with 1 h of incubation at 37°C. All nuclei were stained using 4′,6-Diamino-2-phenylindole (DAPI) (Sigma-Aldrich, St Louis, MO, USA). The infected and non-infected macrophages were washed three times with PBS and analyzed under fluorescence microscopy. All the images were acquired by the IN Cell Analyzer 2000 System (GE Healthcare Bio-Sciences Corp., Piscataway, NJ, USA). Additionally, the percentage of the infected macrophage population and the number of yeast cells per macrophage were determined using Investigator IN Cell 1000 Workstation software (GE Healthcare Bio-Sciences Corp.). This software includes an accurate analysis module that allows reaching reliable results to measure the morphology and the fluorescence intensity of user-defined nuclear and cytoplasmic compartments. Thus, cells can be classified into subpopulations by applying one or more filters, according to one or two user-selectable fluorescence or morphological events. For the analysis, the AMJ2-C11 alveolar macrophages were counted as cells based on some parameters, such as cells fluorescence intensity, nuclei fluorescence intensity, cells area, and nuclei area. To measure yeast cells they were assumed as being “organelles,” and the following parameters were considered, organelles mean area, organelles total area, organelles number per macrophages, organelles fluorescence intensity. The final results were automatically obtained in a worksheet detailing the measures by well, by field, and by cell, regarding the indicated parameters as numerical values. The assay was performed in duplicate.

### Infection rate of *histoplasma capsulatum* in alveolar macrophages detected by flow cytometry

For this assay, AMJ2-C11 macrophage monolayers containing 10^5^ macrophages per well were formed in 24-well plates (TPP®). After, 500 μL (1 × 10^6^ yeasts/mL) of each inoculum of *H. capsulatum* was stained with 10 μM carboxyfluorescein diacetate succinimidyl ester (CFSE) (Invitrogen, Carlsbad, CA, USA) at 37°C for 30 min. Stained *H. capsulatum* strains were added to their respective macrophage monolayer, and the plates were incubated at 37°C, 5 h. After the incubation time, the monolayers were washed three times with sterile PBS, and macrophages were detached at 37°C for 2 min using trypsin-EDTA (Gibco Life Technologies) diluted in PBS. Macrophage suspensions were harvested in Eppendorf tubes and centrifuged at 600 × *g*, 4°C. Supernatants were removed and PBS was added to each Eppendorf tube before cell counting by flow cytometry (BD FACSCanto Becton Dickinson, San Diego, CA, USA). For the analyses, we considered parameters related to the size (size forward scatter—FSC), granularity (granularity side scatter—SSC) and fluorescence of 10,000 cells per tube. The results were determined through the fluorescence intensity (FI) of yeast cells labeled with CFSE as estimated by BD FACSDiva software. Gates of specific population were viewed and analyzed by dot-plot. These data allowed one to determine the percentage of infected alveolar macrophages and discriminate the infectivity of different strains of *H. capsulatum*. Non-infected AMJ2-C11 alveolar macrophages, fluorescein-labeled yeast, and unlabeled yeast were used as negative controls in the assay. Assays were performed in three biological replicates and two technical replicates.

### Comet assay

AMJ2-C11 macrophages, in 24-well plates, were infected with *H. capsulatum* strains EH-315 or 60I using a standardized suspension of 1 × 10^6^ yeasts/mL and incubated at 37°C for 5 h. Non-infected macrophages were used as a negative control. The alkaline version of the comet assay (single cell gel electrophoresis) was performed as described by Singh et al. (1988). Duplicate slides were prepared and stained with ethidium bromide. We screened 50 AMJ2-C11 macrophages per sample with a fluorescence microscope (Carl Zeiss GmbH, Oberkochen, Germany) equipped with a 515–560 nm excitation filter, a 590 nm barrier filter, and a 40 × objective. The level of DNA damage was assessed by an image analysis system (TriTek CometScore, version 1.5; TriTek Corp., Sumerduck, VA, USA), and the DNA percentage in comet tail was obtained for each treatment. Additionally, the percentage of the macrophage population that showed DNA damage was determined.

### TUNEL assay

DNA fragmentation in infected macrophages was evaluated using TUNEL (terminal deoxynucleotidyl transferase dUTP nick-end labeling) staining following the protocol recommended by the manufacturer (Roche Diagnostics, Penzberg, Germany), which has the feature of specific labeling of fragmented DNA sequences that occur during the process of apoptosis. Infection was performed with *H. capsulatum* strains EH-315 and 60I in AMJ2-C11 macrophages cultured in 96-well plates. The macrophages were incubated with a standardized suspension (1 × 10^6^ yeasts/mL) of *H. capsulatum* EH-315 or 60I and infection was allowed for 30 min, 2 h, and 5 h. Non-infected macrophages were used as a negative control. After each incubation time, macrophages were PBS washed and fixed in 4% paraformaldehyde for 1 h at room temperature. Samples were washed three times with cold PBS and incubated with 200 μL permeabilization solution (0.05 M Tris, 0.02 M CaCl2, and 2.5 mg/mL proteinase K) for 15 min at room temperature. After further washing with cold PBS, free reactive sites of the macrophage monolayer on the coverslips were blocked with 200 μL of a solution containing 3% bovine serum albumin and 20% fetal bovine serum in PBS at 37°C for 1 h. Then, the monolayers were washed three times with cold PBS and incubated with the components of the “TUNEL” mixture (dUTP solution containing the enzyme FITC-conjugated and “terminal deoxynucleotidyl transferase”) at 37°C, for 1 h, in a moist chamber under darkness. During the incubation period, the 3′ ends of the apoptotic DNA fragments were incorporated into the FITC-labeled nucleotides. This reaction was catalyzed by terminal transferase. After incubation, three washes were performed with cold PBS, and 100 μL of 1% paraformaldehyde was added per well. Analysis of DNA fragmentation in macrophages was conducted to compare the EH-315 and 60I strains, using non-infected macrophages as a negative control. Images were captured using the IN Cell Analyzer 2000 System for light microscopy and were analyzed by Investigator IN Cell 1000 Workstation software (GE Healthcare Bio-Sciences Corp.). The results were evaluated using as parameter the fluorescence intensity emitted by the nucleus in each condition tested.

### Labeling of the nuclear envelope proteins SUN2, Nesprin2, and Emerin

AMJ2-C11 macrophages, cultured in 24-well plates (TPP®), were infected with a standardized suspension of 1 × 10^6^ yeasts/mL of *H. capsulatum* strains EH-315 or 60I at 37°C for 5 h. Nuclear envelope proteins were marked in either infected or non-infected AMJ2-C11 macrophages (negative control).

Initially, infected and non-infected macrophage samples were fixed with 4% paraformaldehyde, washed with PBS and permeabilized with 0.5% Triton X-100 for 30 min. Then, blocking was performed with 2.5% bovine serum albumin, 1% non-fat milk, and 8% fetal bovine serum. Primary anti-*H. capsulatum* antibody was added for 1 h. Unbound antibodies were removed by washing in PBS, then, Alexa Fluor®594-conjugate goat anti-rabbit IgG (secondary antibody) was added for 1 h. Afterward, in another staining series, anti-SUN2 antibody (Santa Cruz Biotechnology Inc., Heidelberg, Germany), anti-Nesprin2 antibody (Abcam, Cambridge, UK), or anti-Emerin antibody (Abcam) obtained in mice was added as a primary antibody, and macrophage samples were incubated overnight. Unbound antibodies were removed by PBS washing and a secondary Alexa Fluor®488-conjugate goat anti-mouse IgG antibody was added for 1 h. All nuclei were DAPI stained. The infected and non-infected macrophages were then washed three times with PBS and analyzed under confocal laser scanning microscopy (Leica TCS SP5 Confocal Microscopy System). The assay was performed in duplicate.

### Statistical analyses

Data were analyzed using Origin 7.0 (Origin Lab. Corporation, Northampton, MA, USA). ANOVA was used to compare groups in CFU and TUNEL assays with the Bonferroni post-test. *P* was calculated by Student's *t*-test and *P* ≤ 0.001 were considered statistically significant. For the comet assay, the infected and non-infected macrophages (control) were compared using the Kruskal–Wallis test and the associated Dunn post-test with *P* ≤ 0.05 were considered as statistically significant.

## Results

### Infection rate of *histoplasma capsulatum* in AMJ2-C11 alveolar macrophage cell-line detected by CFU

**Figure 1:**
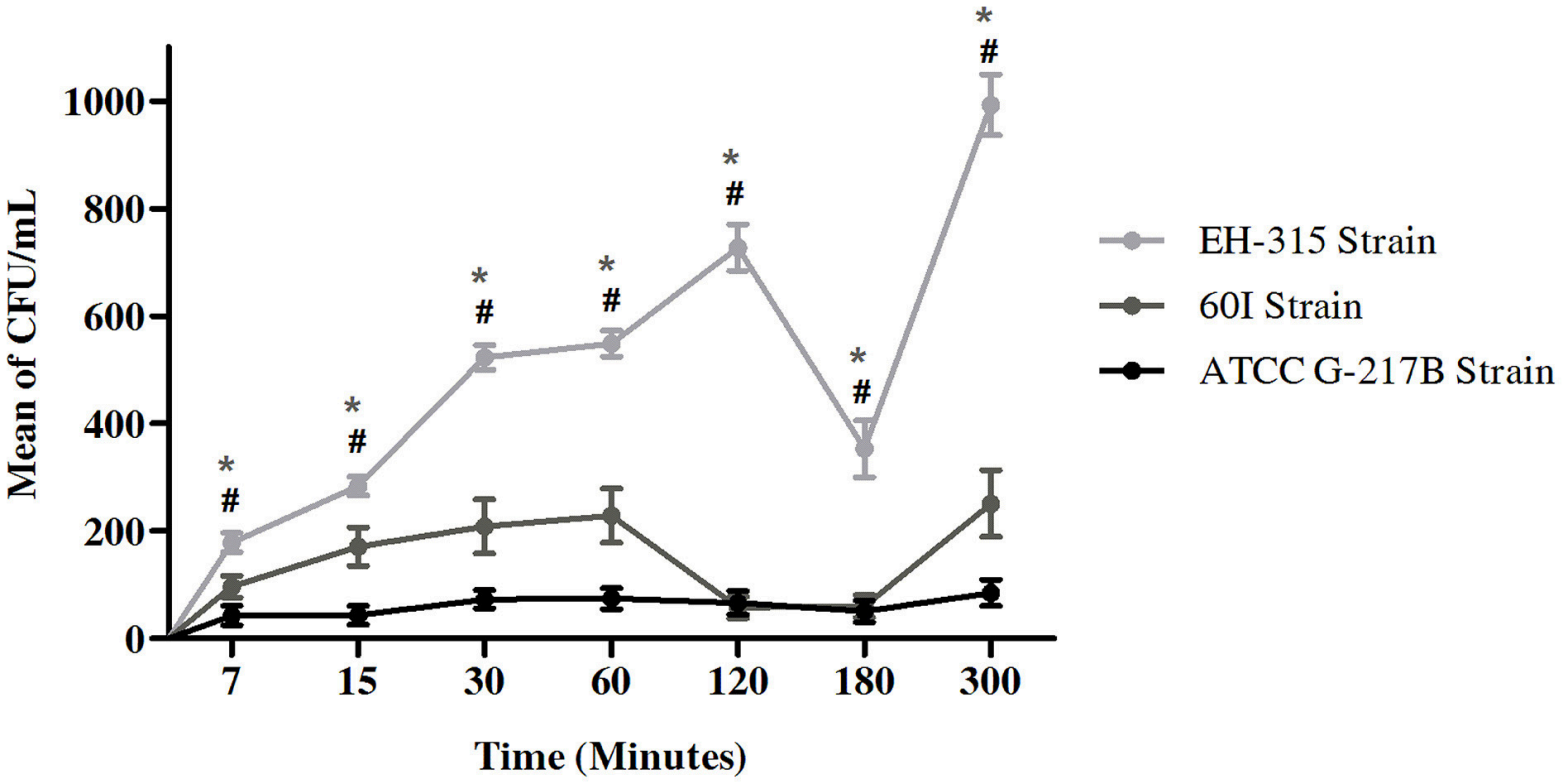
**Infection rate assays of AMJ2-C11 alveolar macrophage with *H. capsulatum*strains G-217B, EH-315, and 60I**. Rates of *H. capsulatum* yeasts were considered using CFU/mL. Scores given are the mean ± S.D. and statistics were performed by Two-way ANOVA with the Bonferroni post-test. ^*^P < 0.001 for EH-315 vs. 60I strain and #*P* < 0.001 for EH-315 vs. G-217B strain.

The infection rate of *H. capsulatum* yeasts was assessed with the *H. capsulatum* reference strain G-217B and strains EH-315 and 60I. As seen in Figure 1, strains EH-315 and 60I developed higher infection rates than the G-217B reference strain. Additionally, the EH-315 strain always exhibited the highest AMJ2-C11 macrophage infection rate at all times studied. Figure 1 shows the number of yeast cells that infected the alveolar macrophages through the CFU/mL counting with these three fungal strains. The results suggested that at 120 min of contact, between alveolar macrophages and strain EH-315, the number of yeast cells in the macrophages increased. At 180 min, the infection rate declined, and it increased again at 300 min. With regard to the strain 60I, the number of yeast cells was increased at 60 min and at 120–180 min, the infection rate declined. Similarly to strain EH-315, the infection rate of strain 60I increased again at 300 min.

### Observation of *histoplasma capsulatum* in AMJ2–C11 macrophages

**Figure 2:**
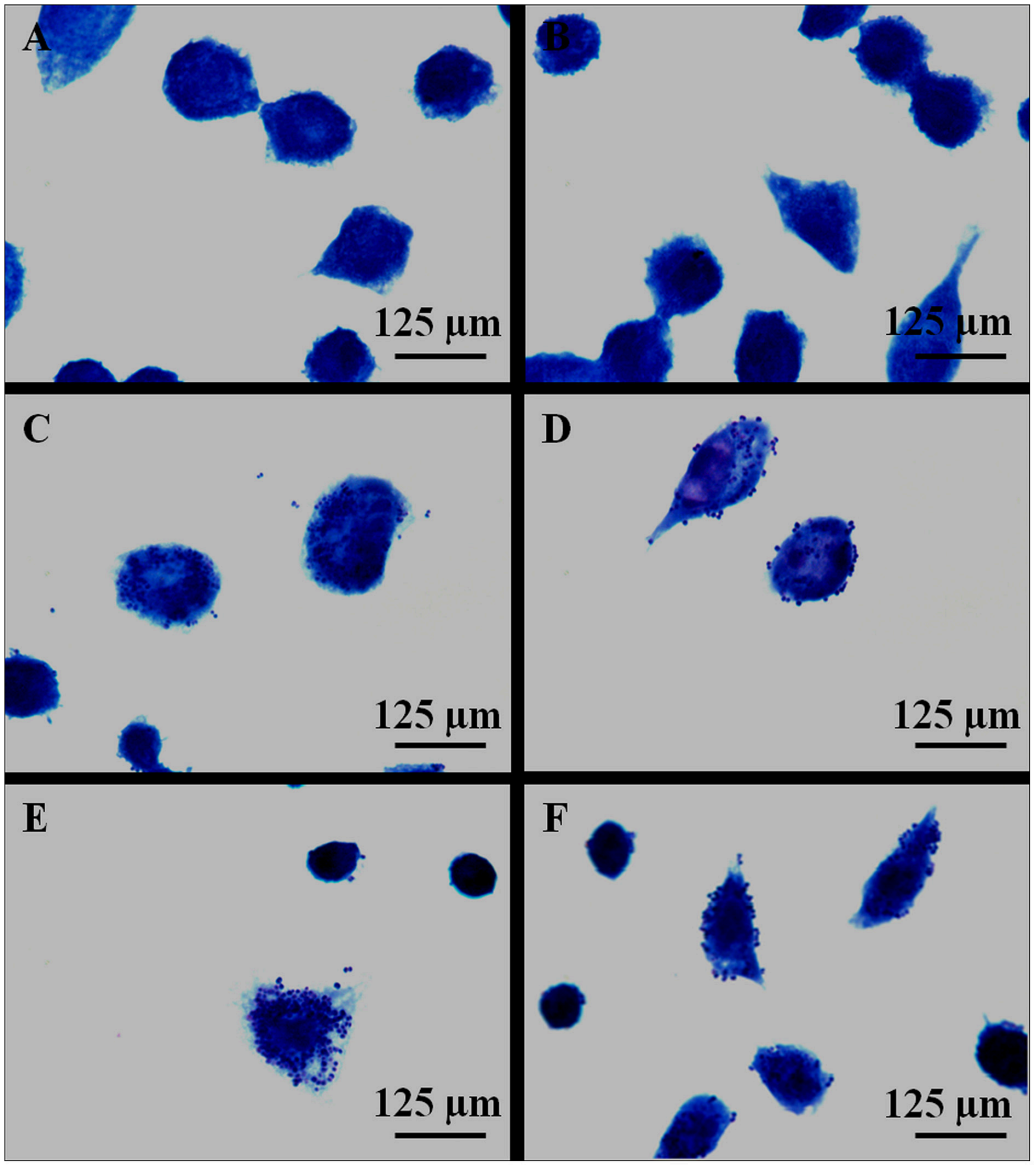
**Giemsa staining of AMJ2-C11 macrophages after 5 h of *H. capsulatum* infection. (A,B)** Control of non-infected macrophages incubated with PBS. **(C,D)** Macrophages infected with *H. capsulatum* strain EH-315. **(E,F)** Macrophages infected with *H. capsulatum* strain 60I. The results are representative of two assays.

Infection of macrophages by *H. capsulatum* yeast (strains EH-315 and 60I) was also evaluated using Giemsa staining. This methodology was very useful for analyzing how yeasts interact with host macrophages (Figure 2). However, this staining did not provide accurate localization of yeast cells within phagocytes.

**Figure 3:**
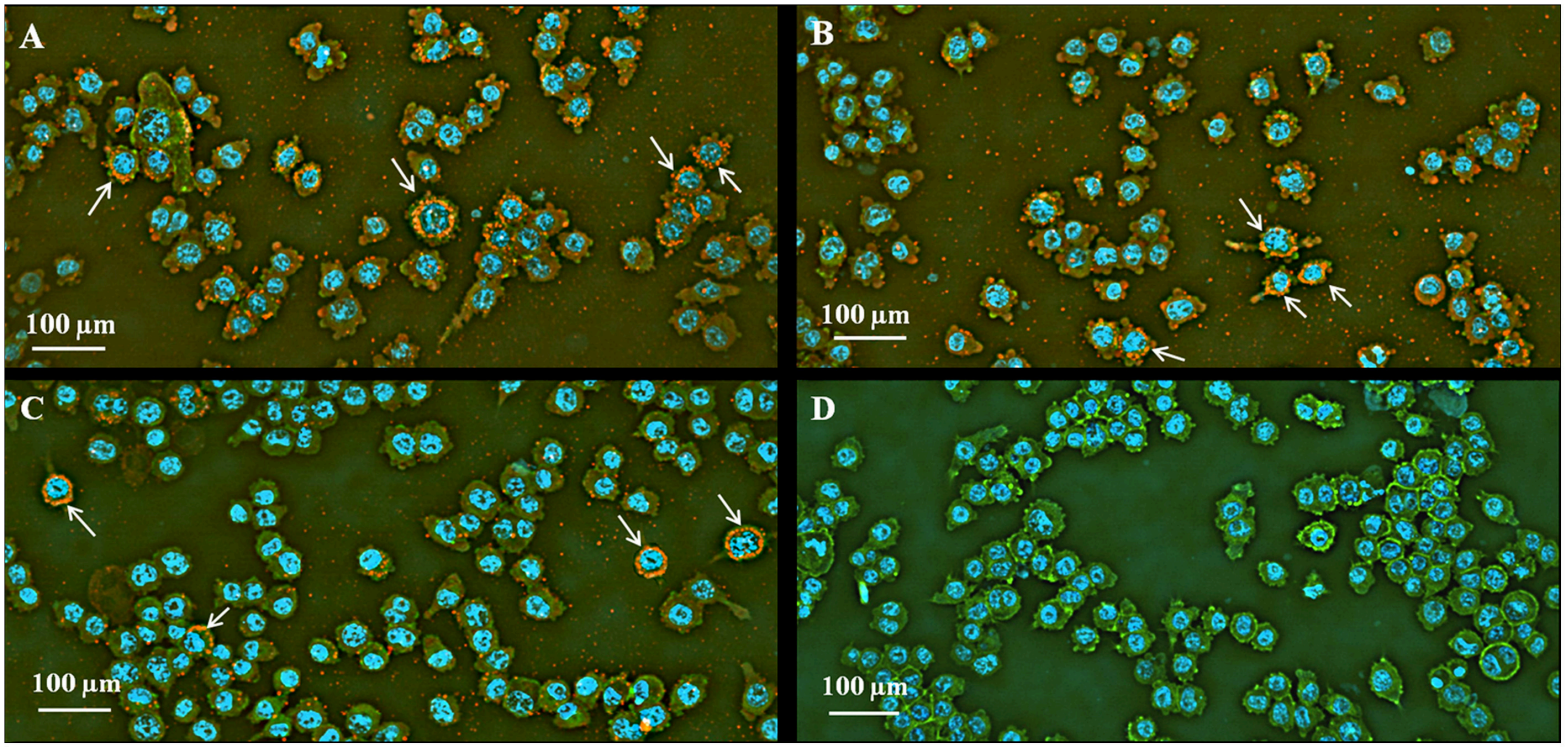
**Effect of *H. capsulatum* yeast cells on the infection of AMJ2-C11 macrophages**. Macrophages infected with *H. capsulatum* yeast cells were incubated at 37°C for 5 h (see details in Section Materials and Methods). **(A,B)** AMJ2-C11 macrophages infected with the EH-315; **(C)** AMJ2-C11 macrophages infected with 60I; and **(D)** non-infected AMJ2-C11 macrophages. Indirect immunofluorescence: FITC-phalloidin in green showing the macrophage cytoplasm; Alexa Fluor®594 in red to yellow staining *H. capsulatum* yeast cells; DAPI in blue staining macrophages nucleus. Images were obtained using IN Cell Analyzer light microscopy. The results are representative of two assays. Arrows indicate the architectural arrangement of yeast-aggregates apparently surrounding the nuclei of the phagocytic cells.

Indirect fluorescence microscopy, using images of the IN Cell Analyzer, revealed several intracellular yeasts in infected macrophages. An interesting finding was detected with this methodology, where intracellular *H. capsulatum* yeast cells aggregated in an architectural shape apparently surrounding the macrophage nucleus, resembling a “crown” (Figure 3). Moreover, additional analyses with Investigator IN Cell 1000 Workstation software showed that strain EH-315 infected 95% of the macrophage population with an infection multiplicity up to 30 cells per macrophage, whereas strain 60I infected 86% of macrophages with up to 24 yeast cells per macrophage.

### Flow cytometry assay

**Figure 4:**
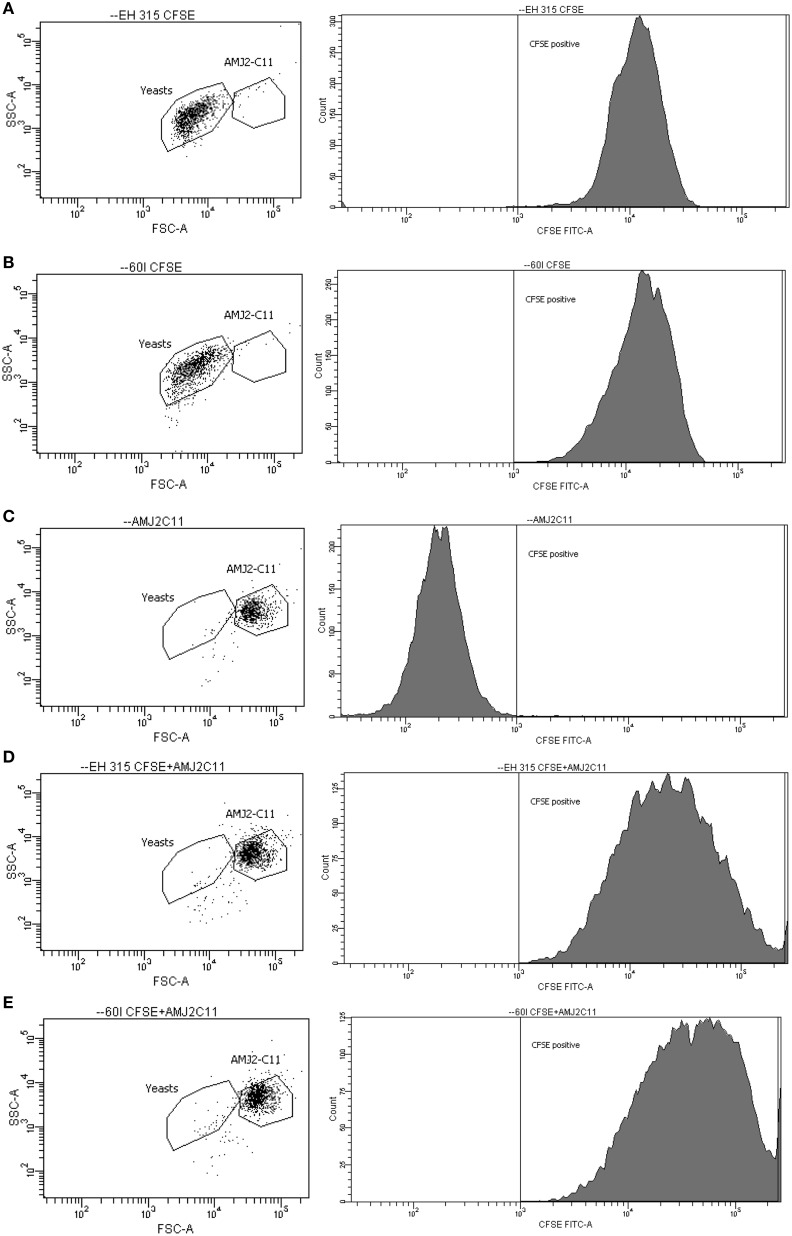
**Dot-plot and histogram profiles of AMJ2-C11 alveolar macrophages and CFSE-labeled *H. capsulatum* yeast cells, at 5 h post-infection. (A,B)** Population of labeled yeast cells (EH-315 and 60I strain, respectively); **(C)** specific gate for AMJ2-C11 alveolar macrophages; and **(D,E)** Infected alveolar macrophages by EH-315 and 60I strain, respectively. The cells were analyzed by flow cytometer BD FACSCanto.

Results were expressed as FI of yeast labeled with CFSE and correspond to the fluorescence data of 10,000 cells per tube. To quantify the percentage of yeast cells bound to or within AMJ2-C11 macrophages, combination of two gates were applied to yeast cells and AMJ2-C11 cell-line (Figure 4). After using these combined gates, immediately the percentage of yeast cells interacting with AMJ2-C11 alveolar macrophages was determined. Regarding the profile of *H. capsulatum* infection in alveolar macrophages, strains EH-315 and 60I showed high infection rates in AMJ2–C11 macrophages. Moreover, both strains have a similar potential for infection because they are able to infect murine alveolar macrophages at rates of 98.34 and 96.52%, respectively, after 5 h of infection. The results represent the average of three independent assays set up in triplicate.

### Comet assay

**Figure 5:**
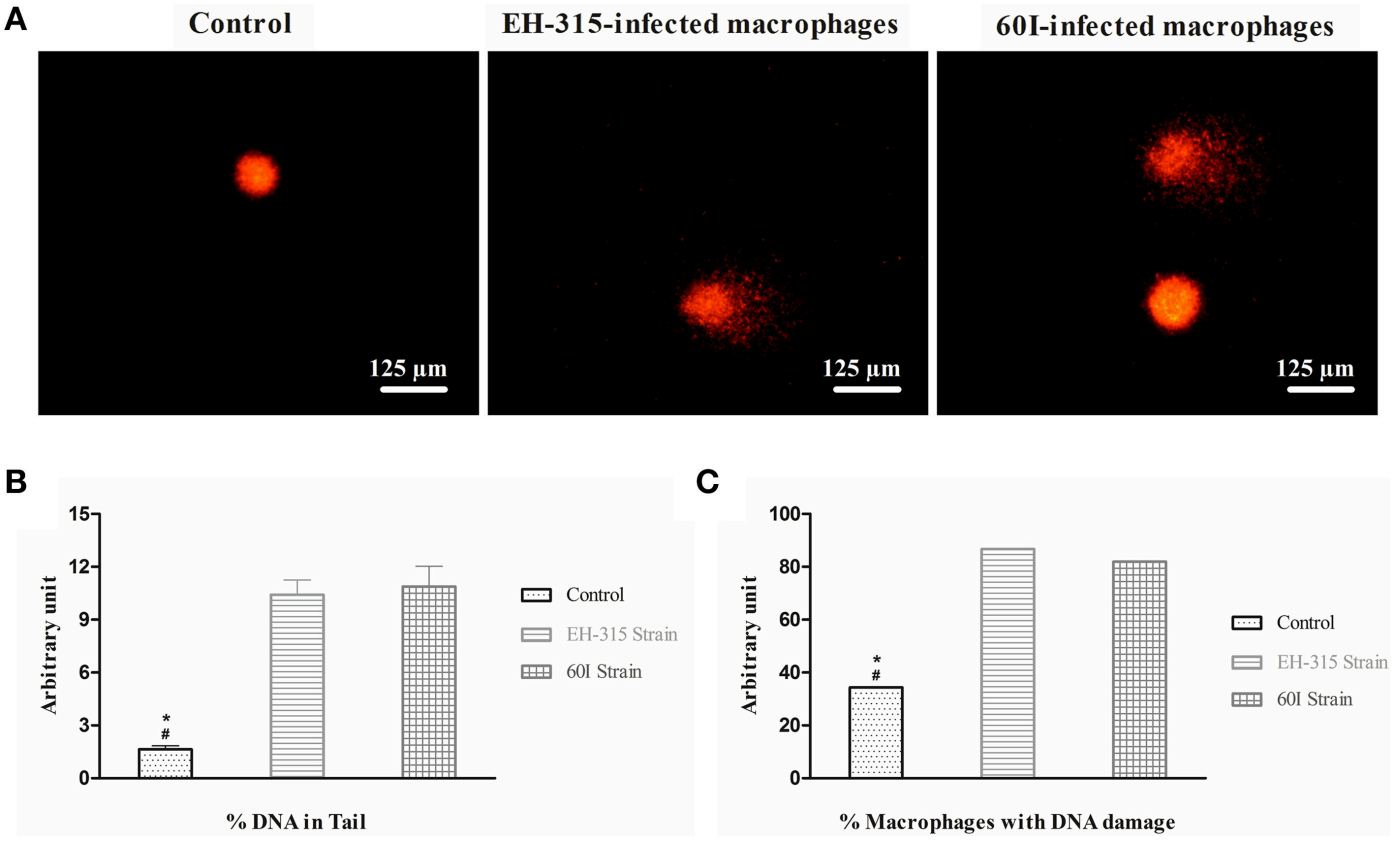
**Comet assay data of AMJ2-C11 macrophages infected with *H. capsulatum* yeasts. (A)** Images were acquired using a fluorescence microscope equipped with a 515–560 nm excitation filter and a 590 nm barrier filter. **(B)** Evaluation of nuclear fragmentation in AMJ2-C11 macrophages infected with *H. capsulatum*. Control (non-infected macrophages). Values are representative of the percentage of fragmented DNA present in the comet's tail. **(C)** Percentage of macrophage population showing DNA damage in infected and non-infected (control) cells. Scores given are the mean ± SD. Data were analyzed by Kruskal-Wallis test with Dunn's post-test. ^*^P < 0.05 when control vs. strain EH-315 was compared; #*P* < 0.05 when control vs. strain 60I was compared.

A similar pattern of DNA fragmentation was observed in infected AMJ2–C11 macrophages by comet assay, when *H. capsulatum* strains EH-315 and 60I were tested. Typical images of the comet assay showing DNA fragmentation in the tail are presented in Figure 5A. In the analysis of macrophage nuclear fragmentation by *H. capsulatum*, DNA damage corresponded to the percentage of DNA in the tail of the comet, and the results demonstrated that, for strains EH-315 and 60I, macrophage DNA damage was 10.67 ± 0.91% and 10.78 ± 1.31%, respectively; whereas 1.75 ± 0.18% of DNA damage was associated with the non-infected macrophages used as a negative control. Significant differences (*P* < 0.05) were found when macrophages infected with each strain were statistically compared with their respective negative controls (Figure 5B).

In addition, the comet assay data also revealed that 86.71% of the macrophage population infected with strain EH-315 showed DNA damage. Similarly, strain 60I induced DNA damage in 81.98% of infected alveolar macrophages. There was no statistically significant difference between the percentages of macrophages undergoing DNA damage induced by the EH-315 or 60I *H. capsulatum* strains. However, significant differences (*P* < 0.05) were found between each macrophage population infected with a fungal strain compared with its respective non-infected control, which revealed 34.41% of DNA damage (Figure 5C).

### TUNEL

**Figure 6:**
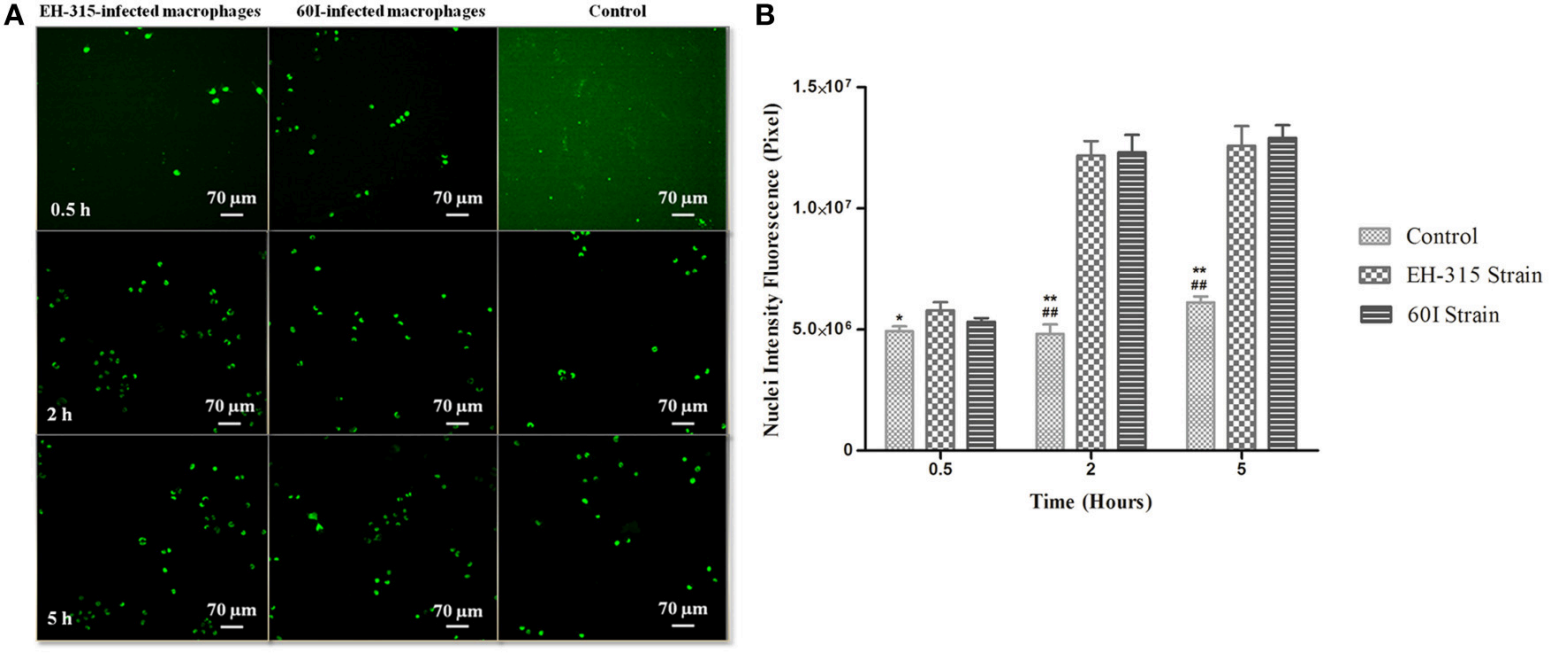
**TUNEL staining of alveolar macrophages after *H. capsulatum* infection. (A)** DNA fragmentation in the nucleus of AMJ2-C11 macrophages infected with *H. capsulatum* yeasts. Images were obtained after 0.5, 2, and 5 h after infection of alveolar macrophages with *H. capsulatum* strains EH-315 or 60I. Infected alveolar macrophages were compared with non-infected macrophages incubated with PBS (control). Each photomicrograph was processed by IN Cell Analyzer light microscopy using a 20X power field. The results are representative of two assays. **(B)** Detection of nuclear fluorescence intensity derived from DNA fragmentation in AMJ2-C11 macrophages infected with *H. capsulatum* strain EH-315 or 60I and non-infected macrophages (control). TUNEL-positive macrophages are represented by the nuclear fluorescence intensity with values derived from analysis of images using Investigator IN Cell 1000 Workstation software. Scores given are the mean ± S.D and statistics were performed by Two-way ANOVA with the Bonferroni post-test. ^*^P < 0.05 and ^**^P < 0.001, control vs. strain EH-315, ##*P* < 0.001, control vs. strain 60I.

Figure 6A shows images obtained by the IN Cell Analyzer for the TUNEL assay that was used to quantify macrophage apoptosis. The measurement of apoptotic nuclei was performed by the release of nuclear fluorescence intensity detected with the TUNEL method; thus, as the fluorescent labeling increases more damage is detected. DNA fragmentation was detected in AMJ2-C11 macrophages infected with *H. capsulatum* strain EH-315 or 60I at 30 min, 2 h and 5 h post-infection. The infection of macrophages with *H. capsulatum*, after 2 and 5 h, resulted in more apoptotic cells than the non-infected controls, and the number of apoptotic nuclei obtained from macrophages infected with each fungal strain was similar, as shown in Figure 6B.

### Labeling of the nuclear envelope proteins SUN2, Nesprin2, and Emerin

**Figure 7:**
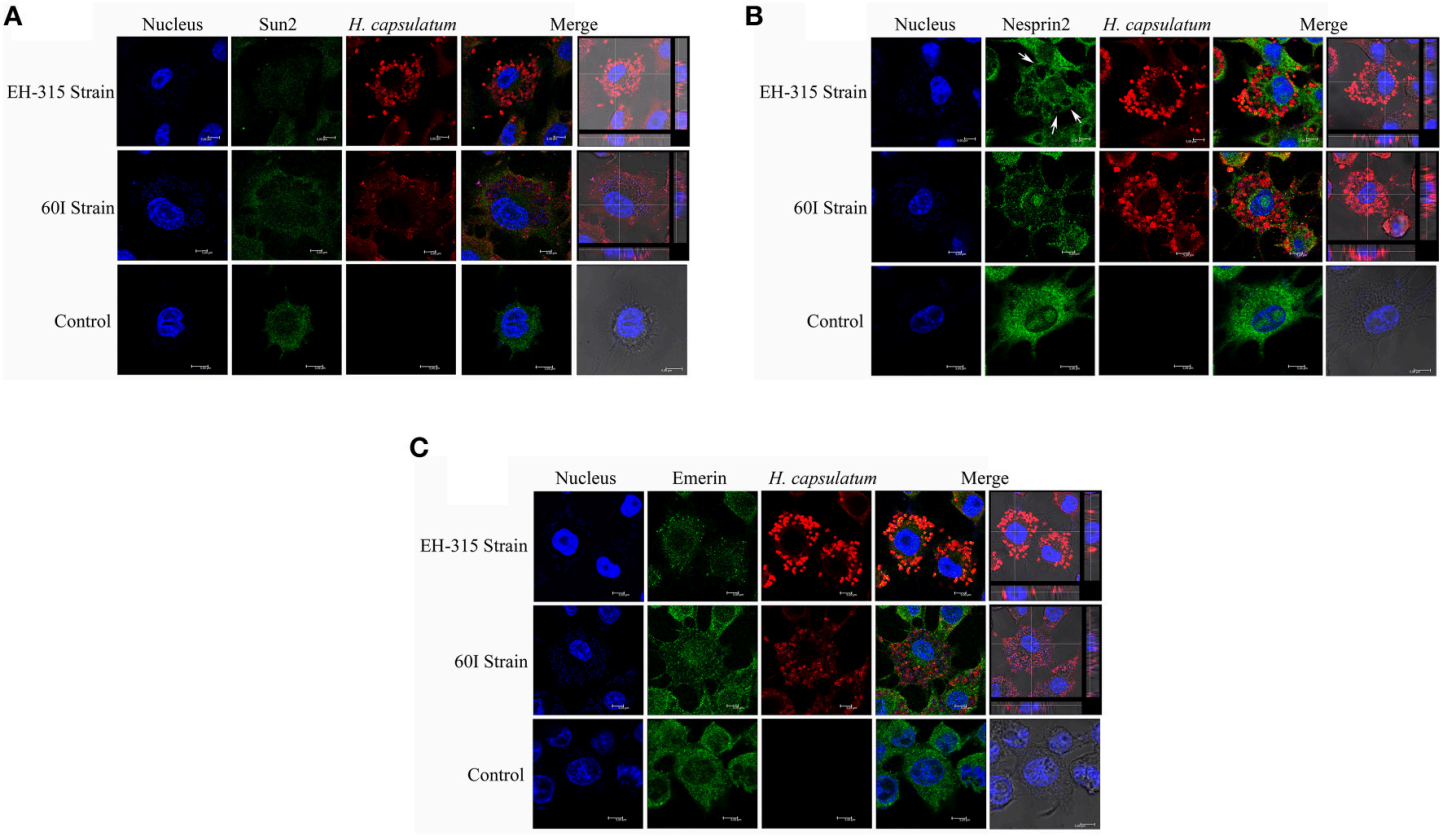
**Labeling of nuclear envelope proteins SUN2, Nesprin2, and Emerin in *H. capsulatum* infected alveolar macrophages**. In Panels **(A–C)** show confocal scanning microscopy images of representative AMJ2-C11 macrophages infected with *H. capsulatum* strains EH-315 and 60I at 37°C for 5 h. Non-infected macrophages were processed as a control. **(A)** AMJ2-C11 stained for SUN2, **(B)** Nesprin2, and **(C)** Emerin as indicated in the figure. The arrows show phagosome formation where several yeast cells are present. DAPI was used for nuclear staining (blue). Alexa Fluor®488 and Alexa Fluor®594 conjugates were used as secondary antibodies to reveal nuclear proteins (green) and yeast cells (red), respectively. Bright field images were merged with Alexa Fluor®594 and DAPI stain with orthogonal z stack sections after 5 h of macrophage-yeast infection. The assay was performed twice.

Confocal microscopy was used to generate 3D images of infected macrophages labeled with anti-SUN2, anti-Nesprin2 and anti-Emerin antibodies. A diffuse distribution of these proteins was found outside on the nuclear envelope, with a similar pattern for non-infected macrophages, as shown in Figure 7. In addition, data from confocal microscopy also indicate that the nuclear fragmentation induced after infection, which was demonstrated by the comet and TUNEL assays, may occur as the result of the architectural conformation displayed by *H. capsulatum*, in which yeast cells appear to surround the macrophage nucleus after 5 h of infection (Figure 7 and Videos S1–S6). Moreover, during infection the formation of large phagosomes within *H. capsulatum*-infected macrophages was noted (Figure 7B). These events were not found in non-infected macrophages.

## Discussion

Interactions of pathogenic fungi with host tissues are essential factors in the pathogenesis of mycoses (Tronchin et al., 2008). *H. capsulatum* infects different host cells, such as neutrophils, macrophages, dendritic cells, and epithelial cells.

The present study demonstrated particular characteristics of the interaction between *H. capsulatum* yeast cells and cultured murine alveolar macrophages. We compared two virulent *H. capsulatum* strains isolated from different sources, EH-315 and 60I, based on their behavior and potential infection for AMJ2-C11 alveolar macrophages. The EH-315 strain developed higher virulence (LD50 3 × 10^5^ yeasts/mL) when compared to the 60I strain (LD50 3 × 10^8^ yeasts/mL) under experimental conditions using an LD50 assay in male BALB/c mice (ML Taylor, personal communication).

We evaluated the ability of the EH-315 and 60I strains to infect alveolar macrophages when compared with the ATCC strain G-217B using CFU analysis of *H. capsulatum* yeast at 7–300 min (5 h) post-infection. The results demonstrated that the three strains of *H. capsulatum* have distinct efficiency for infecting alveolar macrophages. Strain EH-315 developed a better ability to infect macrophages than strains 60I and G-217B. However, regarding the efficacy of the CFU assay, other researchers have described the inconvenience and limitations of this method. According to Berkes et al. (2012), several factors contribute to non-optimize microorganisms plating that routinely reaches only 30% effectiveness for *H. capsulatum*, as the CFU number is generally lower than the number of viable yeasts plated.

Few studies have associated pathogen virulence with the ability to infect host cells or to adhere to abiotic surfaces. Thewes et al. (2008) performed phenotypic screening to compare the SC5314 strain of *Candida albicans*, which is invasive and highly virulent, with the strain ATCC 10231, which is non-invasive and less virulent. Their findings highlight that strain ATCC 10231 caused less damage to fibroblasts and epithelial cells when compared to SC5314. According to those authors, biological properties that influence adherence and invasion to host cells are critical attributes of *C. albicans* in colonization and disease progression, and the results of this study demonstrated that virulence had a direct influence on the ability of this fungus to colonize, damage, and invade host tissues. Furthermore, Sepúlveda et al. (2014) compared *H. capsulatum* strains with distinct genotype and virulence and noted that strain G-217B exhibited delayed response to the virulence effect, as in macrophage damage and cytokine production when compared to other strains. Likewise, Sahaza et al. (2015) highlighted that lung inflammatory responses, in regard to cytokine profile and lung-granuloma formation, varied in intensity and time when two different virulent *H. capsulatum* strains from distinct phylogenetic species, EH-46 (LAm A) and G-217B (NAm 2), were used. Our data corroborated those reported by Sepúlveda et al. (2014) and Sahaza et al. (2015), as strain G-217B showed delayed infection potential against host cells.

In the current study, the infection profile of *H. capsulatum* strains on alveolar macrophages over a period of 5 h was characterized by a variable behavior (increases and decreases) in the yeast infection rate of alveolar macrophages. We hypothesize that this profile occurs as a result of the dynamic interactions between yeast and macrophage membrane receptors.

According to our results, macrophage infections were also monitored at 5 h by Giemsa staining and indirect fluorescence. As we mentioned before, Giemsa staining did not provide well-defined yeast localization within phagocytes, whereas quantification of the macrophage population that was effectively infected with *H. capsulatum* yeasts was successfully achieved by flow cytometric methodology. For these infection assays, the 5 h post-infection time was selected based on a previous kinetic study that showed the largest number of yeast cells interacting with alveolar macrophages at this time-point of infection. Once the CFSE-labeled yeasts had interacted with alveolar macrophages, the percentage of infected cells containing yeast cells was accurately quantified. It is important to note that flow cytometry has been employed to quantify several fungal infections (Chang et al., 1998; Berkes et al., 2012).

*H. capsulatum* is a pathogen that commonly survives within macrophages by developing several intracellular evasion mechanisms (Strasser et al., 1999; Sebghati et al., 2000). Microscopic images obtained by indirect immunofluorescence assays showed a singular pattern of *H. capsulatum* in the infected macrophages under *in vitro* conditions, which was similar for the two fungal strains tested. Interestingly, the yeast cells of both strains were able to form aggregates in the cytoplasm of the infected macrophages with an apparent distribution surrounding the macrophage nucleus after 5 h of infection. These findings could be related to a new strategy for fungal intracellular survival.

Based on the potential for infection displayed by both strains of *H. capsulatum* in alveolar macrophages, it became necessary to evaluate the genotoxic potential of this fungus in AMJ2-C11 macrophages to identify the ability of *H. capsulatum* to induce damage to DNA in host cells. According to Yang et al. (2011), genotoxic agents chemically interact with the genetic material and cause oxidative changes or disruptions in the DNA molecule.

The results of comet and TUNEL assays showed that the two strains of *H. capsulatum* (EH-315 and 60I) caused significant damage to the nuclear DNA of the AMJ2-C11 macrophages after 5 h of infection when compared to non-infected macrophages. Nuclear fragmentation is characterized as a cellular alteration associated with apoptosis (Deepe and Buesing, 2012). In this context, several studies have shown that *H. capsulatum* yeast cells induce apoptosis in different host cell-lines, including macrophages (Allen and Deepe, 2005; Lin et al., 2005; Deepe and Buesing, 2012). According to Das et al. (1999), apoptosis allows the host to develop an effective response against infectious diseases such as tuberculosis.

A study conducted by Del Vecchio et al. (2009) demonstrated the ability of a dimorphic fungus, *Paracoccidioides brasiliensis*, to induce apoptosis in A549 epithelial cells after 24 and 48 h of infection using the TUNEL assay to assess DNA fragmentation. Previous studies have reported that strains of *C. albicans* also induce apoptosis of macrophages after 30 min of infection under *in vitro* conditions. According to the authors, the ability of *C. albicans* to induce apoptosis may modulate a standard anti-inflammatory immune response in the host (Gasparoto et al., 2004). Moreover, polysaccharides of *Cryptococcus neoformans* also induce apoptosis in macrophages under *in vitro* and*in vivo* conditions, compromising the host immune response (Villena et al., 2008).

According to our findings, the apoptosis of infected macrophages could be related to the formation of *H. capsulatum* aggregates that extend throughout the cytoplasm and display a conformational architecture that arrange themselves near to the macrophage nucleus. This conformational aggregation of yeast cells could form in the intracellular environment and remain within the macrophages, causing damage to the nucleus of the host cell and producing DNA fragmentation. This can be explained by the high percentage of infected macrophages with yeast-aggregates after 5 h of infection, which was similar to the percentage of macrophage suffering DNA damage induced by both strains. This new structural arrangement could be associated with the ability of *H. capsulatum* yeasts to prevent elimination by the immune system (Pitangui et al., 2012). The preference of *H. capsulatum* yeast cells to form intracellular aggregates became understandable when a high number of yeasts appeared as linked to each other, during infection of murine alveolar macrophages (Figure 7 and Videos S1–S6).

Confocal microscopy images were used to determine if the effects on host cell nuclei induced by *H. capsulatum* aggregates could change the behavior of the nuclear membrane proteins SUN2, Nesprin2, and Emerin. The analyses revealed that infected macrophages with yeast-aggregates surrounding the macrophage nuclei did not show disruption in the organization of the nuclear lamina that underlies the nuclear envelope, given that the staining of nuclear proteins showed a very similar distribution to that seen in non-infected macrophages. Meinke et al. (2011) reported that disruption of SUN2 or Nesprin2 prevents nuclear movement. In the present study, it was possible to observe diffuse distribution of these proteins outside on the nuclear envelope in both infected and non-infected macrophages.

To date, a few studies have described Nesprin isoforms (Zhang et al., 2007; Morris and Randles, 2010; Randles et al., 2010) as a product generated by the alternative splicing of genes encoding Nesprin1 and Nesprin2. These isoforms vary in size, but they contain a common C-terminal region (Randles et al., 2010) and play important roles in cellular organization, especially in positioning the nucleus and other organelles. Nesprin isoforms appear in different subcellular fractions, including outer and inner nuclear membranes associated with organelles such as mitochondria, Golgi complex, sarcoplasmic reticulum and in the plasmatic membrane, where the isoforms form a network connecting these structures to the actin cytoskeleton (Zhang et al., 2007; Morris and Randles, 2010). Therefore, Nesprin can be found away from the nucleus (Gough et al., 2003), which is consistent with the results obtained in this study, where we observed diffuse presence of this protein in the cytoplasm of infected and non-infected macrophages. However, according to Randles et al. (2010), it is difficult to determine whether other isoforms are also present due to the absence of specific antibodies.

Emerin was also found to be diffuse throughout the cytoplasm of non-infected and *H. capsulatum* infected macrophages. Conversely, a study using host cells infected with herpes simplex virus 1 found an irregular distribution of Emerin, as opposed to a uniform alignment on the nuclear membrane, which appeared like bubbles on the surface of the outer nuclear membrane (Leach et al., 2007). Recently, Ho et al. (2013) reported that Emerin regulates gene expression by modulating actin polymerization in the cytoplasm.

According to our results, it is necessary to emphasize that *H. capsulatum* yeast-aggregates were able to cause damage in the nuclear DNA and induce apoptosis in alveolar macrophages after 5 h of infection. This damage to the DNA of macrophages while the yeast cells are not located inside the core could be a fungus strategy for the facilitation of its persistence throughout the host infection. This finding has never been previously described. Hence, the intracellular arrangement and the occurrence of effects induced by *H. capsulatum* yeast-aggregates during the infection could promote the survival of the pathogen in the hostile conditions of the intracellular environment while also contributing to host tissue damage.

## Author contributions

NS, CS and AF conceived and designed the study. NS, JS, AV, CS, JS, RS and FS performed the experiments and analyzed the data. RS, FS and CS collaborated with reagents/materials/analysis tools. All authors read and approved the final manuscript. NS and AF wrote the paper with contributions from GR, MT and MM.

### Conflict of interest statement

The authors declare that the research was conducted in the absence of any commercial or financial relationships that could be construed as a potential conflict of interest.
