# Supplementary material for: An Intracellular Arrangement of Histoplasma capsulatum Yeast-Aggregates Generates Nuclear Damage to the Cultured Murine Alveolar Macrophages
Source: Front Microbiol. 2016 Jan 11;6:1526. doi: 10.3389/fmicb.2015.01526 (PMC4707385; doi:10.3389/fmicb.2015.01526)
Supplement: Supplementary file 7 [file DataSheet1.DOC]

Supplementary Materials

**An intracellular arrangement of *Histoplasma capsulatum* yeast-aggregates generates nuclear damage to the cultured murine alveolar macrophages**

Nayla de Souza Pitangui^1^, Janaina de Cássia Orlandi Sardi^1^, Aline Raquel Voltan^1^, Claudia Tavares dos Santos^1^, Julhiany de Fátima da Silva^1^, Rosangela Aparecida Moraes da Silva^1^, Felipe Oliveira Souza^1^, Christiane Pienna Soares^1^, Gabriela Rodríguez-Arellanes^2^, Maria Lucia Taylor^2^, Maria José Soares Mendes-Giannini^1^, Ana Marisa Fusco-Almeida^1*^

^1^ UNESP - Univ Estadual Paulista, Departamento de Análises Clínicas, Faculdade de Ciências Farmacêuticas, Araraquara, Brazil ^2^ UNAM - Universidad Nacional Autónoma de México, Departamento de Microbiologia y Parasitologia, Facultad de Medicina, México DF, México

^*^ Correspondence: Ana Marisa Fusco Almeida: [ana.marisa@uol.com.br](mailto:ana.marisa@uol.com.br)

**Video S1.** **3D projection of labeling of nuclear envelope protein SUN2 in *H. capsulatum* infected alveolar macrophages.** Video show confocal scanning microscopy of representative AMJ2-C11 macrophages infected with *H. capsulatum* strain EH-315 at 37°C for 5 h. DAPI was used for nuclear staining (blue). Alexa Fluor®488 and Alexa Fluor®594 conjugates were used as secondary antibodies to reveal nuclear proteins (green) and yeast cells (red), respectively.

**Video S2.** **3D projection of labeling of nuclear envelope protein SUN2 in *H. capsulatum* infected alveolar macrophages.** Video show confocal scanning microscopy of representative AMJ2-C11 macrophages infected with *H. capsulatum* strain 60I at 37°C for 5 h. DAPI was used for nuclear staining (blue). Alexa Fluor®488 and Alexa Fluor®594 conjugates were used as secondary antibodies to reveal nuclear proteins (green) and yeast cells (red), respectively.

**Video S3.** **3D projection of labeling of nuclear envelope protein Nesprin2 in *H. capsulatum* infected alveolar macrophages.** Video show confocal scanning microscopy of representative AMJ2-C11 macrophages infected with *H. capsulatum* strain EH-315 at 37°C for 5 h. DAPI was used for nuclear staining (blue). Alexa Fluor®488 and Alexa Fluor®594 conjugates were used as secondary antibodies to reveal nuclear proteins (green) and yeast cells (red), respectively.

**Video S4.** **3D projection of labeling of nuclear envelope protein Nesprin2 in *H. capsulatum* infected alveolar macrophages.** Video show confocal scanning microscopy of representative AMJ2-C11 macrophages infected with *H. capsulatum* strain 60I at 37°C for 5 h. DAPI was used for nuclear staining (blue). Alexa Fluor®488 and Alexa Fluor®594 conjugates were used as secondary antibodies to reveal nuclear proteins (green) and yeast cells (red), respectively.

**Video S5.** **3D projection of labeling of nuclear envelope protein Emerin in *H. capsulatum* infected alveolar macrophages.** Video show confocal scanning microscopy of representative AMJ2-C11 macrophages infected with *H. capsulatum* strain EH-315 at 37°C for 5 h. DAPI was used for nuclear staining (blue). Alexa Fluor®488 and Alexa Fluor®594 conjugates were used as secondary antibodies to reveal nuclear proteins (green) and yeast cells (red), respectively.

**Video S6.** **3D projection of labeling of nuclear envelope protein Emerin in *H. capsulatum* infected alveolar macrophages.** Video show confocal scanning microscopy of representative AMJ2-C11 macrophages infected with *H. capsulatum* strain 60I at 37°C for 5 h. DAPI was used for nuclear staining (blue). Alexa Fluor®488 and Alexa Fluor®594 conjugates were used as secondary antibodies to reveal nuclear proteins (green) and yeast cells (red), respectively.
